# Evaluation of microscopic observation drug susceptibility assay for diagnosis of multidrug-resistant Tuberculosis in Viet Nam

**DOI:** 10.1186/1471-2334-12-49

**Published:** 2012-03-01

**Authors:** Dang Thi Minh Ha, Nguyen Thi Ngoc Lan, Marcel Wolbers, Vo sy Kiet, Hoang Thi Thanh Hang, Nguyen Hong Duc, To My Huong, Vuong Minh Bach, Nguyen Thi Phuong Thao, Tran Van Quyet, Nguyen Thi Bich Tuyen, Vo Thi Ha, Nguyen Thi Nho, Dai Viet Hoa, Phan Thi Hoang Anh, Nguyen Huy Dung, Jeremy Farrar, Maxine Caws

**Affiliations:** 1 Pham Ngoc Thach Hospital, 120 Hung Vuong, District 5, Ho Chi Minh City, Viet Nam; 2 Wellcome Trust Major Overseas Programme and Oxford University Clinical Research Unit, Hospital for Tropical Diseases, 190 Ben Ham Tu, District 5, Ho Chi Minh City, Viet Nam

**Keywords:** MDR-TB, Tuberculosis, MODS, Diagnosis

## Abstract

**Background:**

Early diagnosis of tuberculosis (TB) and multidrug resistant tuberculosis (MDR TB) is important for the elimination of TB. We evaluated the microscopic observation drug susceptibility (MODS) assay as a direct rapid drug susceptibility testing (DST) method for MDR-TB screening in sputum samples

**Methods:**

All adult TB suspects, who were newly presenting to Pham Ngoc Thach Hospital from August to November 2008 were enrolled into the study. Processed sputum samples were used for DST by MODS (DST-MODS) (Rifampicin (RIF) 1 μg/ml and Isoniazid (INH) 0.4 μg/ml), MGIT culture (Mycobacterial Growth Indicator Tube) and Lowenstein Jensen (LJ) culture. Cultures positive by either MGIT or LJ were used for proportional DST (DST-LJ) (RIF 40 μg/ml and INH 0.2 μg/ml). DST profiles on MODS and LJ were compared. Discrepant results were resolved by multiplex allele specific PCR (MAS-PCR).

**Results:**

Seven hundred and nine TB suspects/samples were enrolled into the study, of which 300 samples with DST profiles available from both MODS and DST-LJ were analyzed. Cording in MODS was unable to correctly identify 3 Mycobacteria Other Than Tuberculosis (MOTT) isolates, resulting in 3 false positive TB diagnoses. None of these isolates were identified as MDR-TB by MODS. The sensitivity and specificity of MODS were 72.6% (95%CI: 59.8, 83.1) and 97.9% (95%CI: 95.2, 99.3), respectively for detection of INH resistant isolates, 72.7% (95%CI: 30.9, 93.7) and 99.7% (95%CI: 98.1, 99.9), respectively for detecting RIF resistant isolates and 77.8% (95%CI: 39.9, 97.1) and 99.7% (95%CI: 98.1, 99.9), respectively for detecting MDR isolates. The positive and negative predictive values (PPV and NPV) of DST-MODS were 87.5% (95%CI: 47.3, 99.6) and 99.3% (95%CI: 97.5, 99.9) for detection of MDR isolates; and the agreement between MODS and DST-LJ was 99.0% (kappa: 0.8, *P *< 0.001) for MDR diagnosis. The low sensitivity of MODS for drug resistance detection was probably due to low bacterial load samples and the high INH concentration (0.4 μg/ml). The low PPV of DST-MODS may be due to the low MDR-TB rate in the study population (3.8%). The turnaround time of DST-MODS was 9 days and 53 days for DST-LJ.

**Conclusion:**

The DST-MODS technique is rapid with low contamination rates. However, the sensitivity of DST-MODS for detection of INH and RIF resistance in this study was lower than reported from other settings.

## Background

The worldwide occurrence of multidrug-resistant tuberculosis (MDR-TB) has been documented by the World Health Organization (WHO), with estimates of nearly half a million cases annually, and 150,000 deaths [1]. MDR-TB is caused by *Mycobacterium tuberculosis *(*M. tuberculosis*) which is resistant to at least the two most powerful TB drugs isoniazid (INH) and rifampicin (RIF). In addition to the high costs, long duration of treatment and the lack of randomized controlled trials for optimal regimens, a major barrier to control of MDR TB is the lack of laboratory diagnostic capacity in high-TB burden settings. Major initiatives are now under way to scale-up capacity for both *M. tuberculosis *culture and drug susceptibility testing (DST) [2]. According to WHO, of 27 high MDR-TB burden countries, only 22 countries had a National Reference Laboratory in 2008. Of 572 laboratories performing drug susceptibility testing (DST), only half participated in external quality assurance [1].

Recently, the documentation in over 50 countries of extensively drug-resistant tuberculosis (XDR-TB), defined as MDR-TB plus resistance to a fluoroquinolone and at least one second-line injectable agent (amikacin, kanamycin or capreomycin) [1] has further emphasized the need to scale up detection and treatment of MDR TB. In HIV co-infected individuals the classical smear diagnostic test has very low sensitivity and the need for enhanced culture has further urgency in settings with HIV co-epidemics; late diagnosis and treatment contributes to high mortality rates and on-going transmission in this population [3]. Classical DST for *M. tuberculosis *on solid media requires 6-8 weeks as the sputum sample must first be cultured and then regrown on drug-containing media. In 2007, WHO recommended the use of liquid culture and DST in low and middle-income countries as a step-wise approach to scale up TB and MDR-TB diagnosis and management [4,5]. Automated liquid culture allows turn-around times of 20-28 days when indirect DST is performed but the automated equipment is expensive for most high-burden settings and cheaper alternative methods may be easier to implement. Other phenotypic techniques have been also been developed. Nitrate reductase assay (NRA), a solid media DST technique which is based on the ability of *M. tuberculosis *to reduce nitrate to nitrite has also been endorsed by WHO [6,7]. Although NRA can be performed on both culture isolates and specimens, more evidence is required regarding the accuracy of NRA applied directly on specimens. Colorimetric methods are based on the reduction of an indicator dye which is added to the culture medium after *M. tuberculosis *has been inoculated with or without antibiotics. The growth of a resistant isolate is detected by a change in colour of the indicator, which is directly proportional to the number of viable mycobacteria in the medium. A review of published data concluded that the sensitivity and specificity of this method are 89% and 100%, respectively, and the result is available in 7-14 days [7]. However, colorimetric assays are performed on culture isolates so a primary isolation is needed which takes approximately 2-4 weeks. The Thin Layer Agar (TLA) assay has also shown promise with for detection of RIF and INH resistant isolates [8], however the WHO strategic technical advisory group (STAG) concluded that there is currently insufficient evidence to recommend the use of TLA for MDR-TB detection and further evaluation is required [6].

Molecular line probe assays (LPA) for rapid detection of MDR-TB, which are based on reverse hybridization technology have been endorsed by WHO in 2009 [9]. With sensitivity and specificity of over 90% and 99%, respectively against the conventional DST method [10], LPA are an alternative rapid and accurate DST method but are only reliable in smear positive cases. Unfortunately, the cost and the requirement for relatively complicated technology and infrastructure in addition to well-trained staff have limited the use of LPA in high TB burden countries where the need is greatest. Xpert MTB/RIF (Cepheid, USA), is a novel real-time PCR based technique which detects *M. tuberculosis *and rifampicin resistance mutations directly from sputum samples. In evaluations in South Africa, the test showed sensitivity and specificity for *M. tuberculosis *detection of 98% and 99%, respectively and correctly identified 97.6% isolates with RIF resistant isolates and 98% of rifampicin susceptible isolates compared with phenotypic DST [11]. However, some false-positive RIF resistance results were initially reported [11,12]. The Ct ratio threshold has been revised but it is not yet clear if this has eliminated false-positive results for RIF resistance. Furthermore, the cost remains relatively high for use in developing countries at the present time, with FIND negotiated pricing of 18USD per test cartridge [13]. Therefore, no single test currently available provides all the characteristics of an ideal test for rapid diagnosis of MDR-TB, which would be rapid, low-cost, easy to perform, and highly sensitive and specific. Microscopic Observation Drug Susceptibility (MODS) assay, is a direct rapid DST test which has been evaluated for MDR screening from clinical specimens. Previous studies have shown it an accurate, feasible and low-cost test that is promising for use in high burden countries for early diagnosis of MDR TB [14-16]. Procedures for quality assurance of the test are still in development [17] and the accuracy of the test when adopted by non-expert groups needs to be confirmed [18]. This technique is now endorsed by WHO for use in resource-limited settings as an interim solution to increase TB case detection [6,9].

Viet Nam is a high TB burden country with steeply rising rate of HIV-TB co-infection [19]; 8% of newly diagnosed TB patients in 2007 were HIV infected [20]. The prevalence of MDR-TB among new TB cases and retreatment cases in 2007 were 2.7% and 19%, respectively. Importantly, XDR-TB has been detected in Viet Nam [21]. These MDR-TB and XDR-TB cases are the most urgently in need of diagnosis because they have the highest morbidity and mortality yet effective diagnosis is not widely available. We conducted a study to evaluate the accuracy of MODS in early diagnosis of MDR-TB in new TB suspects in Viet Nam.

## Methods

### Enrollment

All patients suspected of tuberculosis, who were newly presenting to the Out Patient Department (OPD) at Pham Ngoc Thach Hospital from August to November 2008 were enrolled into the study. Exclusion criteria were an age < 16 years of age or a prior dose of TB therapy.

Data on demographic features, TB history and HIV status were prospectively collected on a standard case report form. Samples were collected as per routine care as deemed appropriate by the treating physician. No additional samples were collected as part of this study and only the first sputum sample of each patient was evaluated.

### Ethics

The protocol was approved by the Institutional Review Board (IRB) at Pham Ngoc Thach Hospital and the Health Services of Ho Chi Minh City. Individual informed consent was not sought because the study was conducted on routine samples only and it did not involve any intervention, additional samples or change in patient management. A patient consent waiver was approved by the IRB of Pham Ngoc Thach Hospital.

### Sample processing

Sputum samples were homogenised and decontaminated by Sputaprep (NaOH-NALC 2%) manufactured by Nam Khoa Company, Viet Nam prior to testing, as previously described [22]. The processed sample was aliquoted into 3 parts for direct DST by MODS, MGIT culture and LJ culture. A positive MGIT or positive LJ culture from each sample was used for 1% proportional DST method (indirect DST method).

### Direct DST by MODS (DST-MODS)

For each processed sample, 2 drug-free wells (control wells), 1 INH containing well and 1 RIF containing well were set up. In brief, the MODS media was prepared with 5.9 g Middlebrook 7H9 broth (Difco, Sparks, MD), 3.1 ml glycerol and 1.25 g bacto casitione (Difco, USA) in 880 ml distilled water. This media was autoclaved, filtered and stored in 4.5 ml tubes at 4°C. Each new batch of media was tested for sterility by incubating one aliquot at 37°C for 1 week. Before use, 0.5 ml OADC (BD), 0.5 ml processed sample and 100 μl PANTA antibiotic (BD) were added into each 4.5 ml tube. Nine hundred microlitres of the suspension was then transferred to each of four wells in a 48 well-plate as described above. Next, 100 μl distilled water was added into the control wells. Finally, 100 μl INH 4 μg/ml (Sigma) or 100 μl RIF 10 μg/ml (Sigma) was added to the INH-containing well and RIF-containing well, respectively. The final concentrations of OADC and PANTA in each well were 10% and 20 μl/ml, respectively. The drug concentrations in each well were 0.4 μg/ml for INH and 1 μg/ml for RIF. One susceptible isolate (H37Rv), one INH-resistant clinical isolate and one RIF-resistant clinical isolate were inoculated to the first plate each day. Resistant control isolates are well-characterised clinical isolates from Pham Ngoc Thach laboratory used as routine controls for all DST procedures. A McFarland 0.5 (approximately 10^4 ^CFU/ml) suspension of each isolate was made and diluted 100-fold (10^2 ^CFU/ml). A 0.5 ml volume of the final suspension was used as the inoculum.

The plate was incubated at 37°C, and the results were recorded on alternate days from day 5 to day 15 and twice a week from day 16 to 1 month. Any cord formation in at least one control well was recorded as a positive MODS culture. If there was any cord formation in both control wells, the drug containing wells were read. If cords were detected in only one control well, MODS-DST was recorded as uninterpretable for technical analysis. Any isolate with growth in both the control and drug-containing wells was recorded as resistant. If growth was observed in control wells but not in the drug-containing wells, a susceptible result was recorded for the relevant drug.

### MGIT culture

All processed samples were aliquoted for MGIT culture following the protocol of Becton Dickinson (BACTEC™ MGIT™ 960 Mycobacerial Detection System) [23].

### Lowenstein-Jensen culture (LJ culture)

All processed samples were cultured on LJ. One hundred microlitres of processed sample was inoculated onto a LJ slant and incubated at 37°C. Results were read weekly from day 21 after inoculation following routine standard operating procedure of Pham Ngoc Thach hospital. The presence of at least one colony with rough shape was recorded as positive culture.

### Subculture on LJ

All cultures positive by MGIT or LJ were subcultured on LJ (Becton Dickinson) for indirect DST, standard biochemical identification (Niacin and Nitrate) and archiving [24]. Mycobacteria Other Than Tuberculosis (MOTT) identified by biochemical tests were confirmed by LiPA MYCOBACTERIA assay (Innogenetics, Belgium).

All cultures positive by MODS (control wells) were subcultured on LJ in duplicate for DNA extraction [25] and archiving [24].

If DNA extraction from MODS isolates failed due to contamination or failure to grow on subculture, DNA extraction from positive MGIT or LJ was performed, if available.

### Indirect DST test-1% proportional DST method (DST-LJ)

Indirect phenotypic DST method was performed at the Reference TB laboratory at Pham Ngoc Thach Hospital, which is accredited by the WHO reference TB laboratory of Western Pacific region (Adelaide, Australia). Indirect DST was performed for all positive MGIT or LJ isolates on Isoniazid 0.2 μg/ml, Streptomycin 4 μg/ml, Rifampicin 40 μg/ml, Ethambutol 2 μg/ml and Pyrazinamide 200 μg/ml according to WHO guidelines [26].

### Multiplex Allele Specific PCR (MAS-PCR) for detection of Isoniazid and Rifampicin resistance mutations

MAS-PCR was performed as previously described to detect mutations in the Rifampicin Resistance Determining Region (RRDR) of the *rpoB *gene at codons 516 (D516V/A/G), 526 (H526D/F/L/R/S/Y/Q/N) and 531 (S531L) [27] and mutations in *katG *(S315T) and inhA (C-15 T) genes for INH resistance [28].

The RRDR region of *rpoB *was sequenced for RIF-discrepant isolates with no RRDR mutation identified by MAS-PCR [27].

In this study, MAS-PCR was used to confirm INH and RIF resistance in isolates classified as susceptible by MODS and resistant by LJ, or vice versa. MAS-PCR cannot be used to 'rule-out' true resistance because approximately 5% and 20% of RIF and INH resistant isolates, respectively will not have mutations in the targeted gene sites [29].

### Spoligotyping

Spoligotyping was performed according to the standard international Spoligotyping protocol [7] for all cultures positive by MODS (n = 329). If MODS was contaminated during subculture from MODS to LJ for DNA extraction (n = 24) or MODS was negative but MGIT positive (n = 36), cultures positive by MGIT were used for spoligotyping. Spoligotyping was used to support the screening of cross-contamination (data not shown). Cross-contamination of MGIT was not addressed in this study due to resource limitations.

### Statistics

Detection rates for MODS, MGIT and LJ were summarized and compared between methods using McNemar's test. The accuracy of MODS for diagnosis of TB was then assessed using MGIT and LJ as the gold-standard reference test, i.e. a sample was defined as positive by the reference test if either MGIT or LJ (or both) were positive. The accuracy of MODS for drug-susceptibility testing was assessed in samples with a valid drug susceptibility test result by both MODS and DST-LJ. The gold-standard reference test was the DST-LJ result. We also summarized agreement between DST-MODS and DST-LJ as raw agreement and by Cohen's kappa. Confidence intervals for accuracy measures (sensitivities, specificities, positive and negative predictive values) were calculated according to the method of Pearson and Clopper. Finally, we compared the time to a positive test for MGIT and MODS, respectively, in samples positive by both methods using the Wilcoxon signed rank test and visualized it using the empirical cumulative distribution.

All reported confidence interval are two-sided 95% confidence intervals and *p*-values ≤ 0.05 were regarded as statistically significant. All analyses and graph were performed with Stata version 9 (Statacorp, Texas, USA).

Cross-contamination was investigated using spoligotyping. Identification of H_37_Rv from any well except the positive control well was considered as a cross-contamination event. Cross contamination was suspected if at least one control well was positive by DST-MODS and both MGIT and LJ culture were negative for that sample. In these cases if spoligotyping showed the isolate was unique on the MODS plate processed that day, contamination was discounted. If the isolate showed a spoligotype identical to another isolate on the same plate, contamination could not be ruled-out and we therefore report the maximum possible cross-contamination rate.

## Results

### Study population and demography

Seven-hundred and nine patients clinically suspected of tuberculosis were enrolled into the study. The median age was 39 years (IQR: 27-53 years). The male:female ratio was 1.7 (n = 447/262). HIV status was unknown for the majority of the patients (99.0%, n = 702/709) and only one patient was recorded as HIV positive.

### Accuracy of MODS culture for *M. tuberculosis* detection against MGIT culture or LJ culture as the gold standard

In 709 sputum samples sent for TB diagnosis, the detection rates of MODS, MGIT and LJ were 50.5% (n = 358/709, 95%CI: 46.7, 54.2), 51.6% (n = 366/709, 95%CI: 47.9, 55.3) and 44.4% (n = 315/709, 95%CI: 40.7, 48.1), respectively. No significant differences in detection rates were found between MODS and MGIT (*P *= 0.80), MODS and LJ (*P *= 0.17) or MGIT and LJ (*P *= 0.11).

There were 373/709 (52.6%) samples positive by either MGIT or LJ culture. The accuracy of MODS in diagnosis of TB is presented in Table 1.

**Table 1:** Sensitivity, specificity, positive predictive value and negative predictive value of MODS in detection of *M.tuberculosis *and INH or RIF resistance

	Sensitivity % (n = x/y) 95%CI	Specificity % (n = x/y) 95%CI	PPV % (n = x/y) 95%CI	NPV % (n = x/y) 95%CI
MODS diagnosis of TB	89.0 (332/373) [85.4, 91.9]	92.3 (310/336) [88.9, 94.9]	92.7 (332/358) [89.5, 91.5]	88.3 (310/351) [84.5, 91.5]

MODS for detection of INH resistant isolates	72.6 (45/62) [59.8, 83.1]	97.9 (233/238) [95.2, 99.3]	90.0 (45/50) [78.2, 96.7]	93.2 (233/250) [89.3, 95.9]

MODS for detection of RIF resistant isolates	72.7 (8/11) [39.0, 93.9]	99.7 (288/289) [98.1, 99.9]	88.9 (8/9) [51.8, 99.7]	98.9 (290/292) [97.5, 99.9]

MODS for detection of MDR isolates	77.8 (7/9) [39.9, 97.1]	99.7 (290/291) [98.1, 99.6]	87.5 (7/8) [47.3, 99.6]	99.3 (290/292) [97.5, 99.9]

PPV: Positive predictive value

NPV: Negative predictive value

### Detection of drug-resistance

#### Drug-resistant isolates detected by 1% proportion method (DST-LJ-the gold standard method) (n = 364)

Although there were 373 samples positive by either MGIT or LJ, 9 samples were initially identified as MOTT by biochemical identification tests (3 *M. fortuitum*, 3 *M. chelonae and 3 unspeciated MOTT) *and therefore DST-LJ was not done for these samples. Of these 9 samples, one sample was MODS negative, one sample was contaminated by fungi on MODS culture and seven samples were culture positive by MODS but could not be differentiated from *M. tuberculosis *complex by cording observation. We subsequently performed the LiPA MYCOBACTERIA assay for the remaining seven isolates. The line probe assay identified four of these isolates as *M. tuberculosis*, all of which were susceptible to both RIF and INH by MODS-DST. The remaining three isolates were identified as *M. kansasii, M. chelonae *(both susceptible to RIF but resistant to INH by MODS) and *M. fortuitum *(susceptible to both RIF and INH by MODS). As a result, 364/373 samples with DST results by 1% proportion method were available for analysis. The multidrug resistant isolates (MDR-TB) account for 3.8% (n = 14/364) of cases. Isoniazid and Rifampicin monoresistance were detected in 3.8% (n = 14/364) and 0.3% (n = 1/364) of samples, respectively.

### Study population for the evaluation of DST-MODS

**Figure 1:**
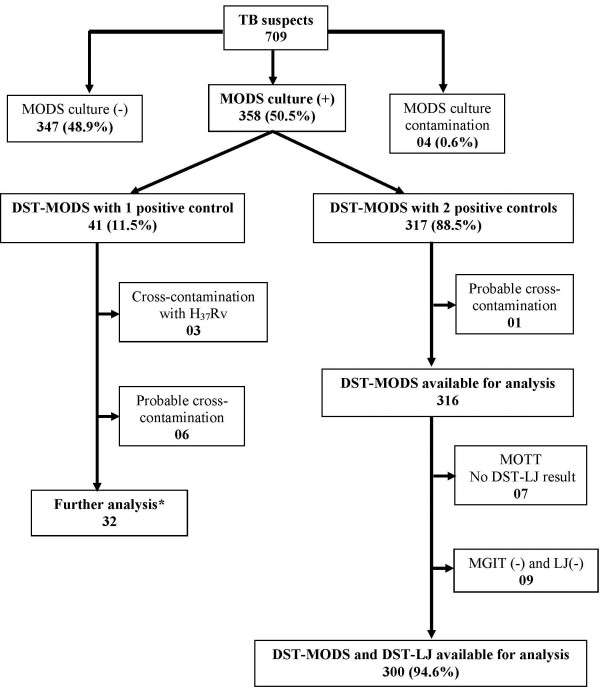
**Recruitment flow chart**. MOTT: Mycobacteria Other Than Tuberculosis. DST-MODS: Drug susceptibility testing done by MODS. DST-LJ: Drug susceptibility testing done by 1% proportional method on LJ media. (*) Analysis based on DST/LJ and MAS-PCR because DST/MODS results were recorded as uninterpretable

Of the 358 samples positive by MODS culture, 88.5% (n = 317/358) had two control wells (drug-free wells) positive by DST-MODS and 94.6% (n = 300/317) of them were eligible for final DST analysis between DST-MODS and DST-LJ (Figure 1). 5.4% (n = 17/317) samples were excluded from analysis because of probable cross-contamination by MODS (n = 1) and no DST-LJ results (n = 16). The remaining positive MODS samples (11.5%, n = 41/358) only had one control well positive by MODS and were analyzed separately.

### DST-MODS analysis (n = 300)

#### DST-MODS against DST-LJ as the gold standard

**Figure 2:**
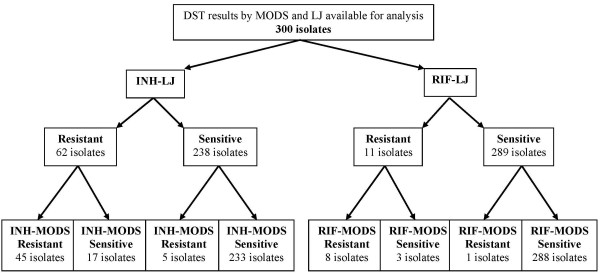
**Drug susceptibility testing results of 300 isolates by MODS and LJ for Rifampicin and Isoniazid**. RIF: Rifampicin, INH: Isoniazid, LJ: proportional DST method on LJ medium, MODS: microscopic observation drug susceptibility assay

Direct drug susceptibility testing results on MODS were compared with indirect DST on LJ as the gold standard for 300 samples (Figure 2). DST-MODS detected INH, RIF and MDR resistant isolates at 16.7% (n = 50/300), 3% (n = 9/300) and 2.7% (n = 8/300), respectively. The accuracy of MODS in detection of INH and/or RIF resistant isolates is shown in Table 1. The agreement between DST-MODS and DST-LJ were 92.7% (n = 278/300, 95%CI: 89.1, 95.3, kappa: 0.75, *P *< 0.001) for detection of INH resistant isolates, 98.7% (n = 296/300, 95%CI: 96.6, 99.6, kappa: 0.79, *P *< 0.001) for RIF resistant isolates and 99% (n = 297/300, 95%CI: 97.1, 99.7, kappa: 0.80, *P *< 0.001) for detection of MDR isolates.

#### Resolution of discrepant results

There was 25/300 isolates with discrepant DST results between MODS and LJ for INH, RIF or both INH and RIF (Table 2). Of which, 3 samples were discrepant for RIF resistance only, 21 samples were discrepant for INH resistance only and 1 sample was discrepant for both INH and RIF resistance.

**Table 2:** Discrepant DST results between MODS and LJ resolved by MAS-PCR

Isolate N0	DST-MODS		DST-LJ		MAS-PCR (mutation point)		Remarks
		
	RIF 1 μg/ml	INH 0.4 μg/ml	RIF 40 μg/ml	INH 0.2 μg/ml	RIF resistant mutation	INH resistant mutation	
1	R	R	S	S	None	R (katG)	MDR by MODS
2	S	R	R	R	_		RIF-MODS resistant on day 20. MDR by LJ. *rpoB *sequencing: synonymous mutation (H445C, CAC > TGC)
3	S	S	R	S	None	None	
4	S	R	R	R	R (531)		RIF-MODS resistant on day 20. **MDR by LJ**
5	S	R	S	S		None	
6	S	R	S	S		None	
7	S	R	S	S		None	
8	S	R	S	S		None	
9	S	S	S	R		R (katG)	INH-MODS resistant on day 30
10	S	S	S	R		None	INH-MODS resistant on day 30
11	S	S	S	R		R (katG)	INH-MODS resistant on day 40
12	S	S	S	R		R (inhA)	
13	S	S	S	R		R (inhA)	
14	S	S	S	R		R (inhA)	
15	S	S	S	R		R (katG)	
16	S	S	S	R		R (inhA)	
17	S	S	S	R		R (katG)	INH-MODS resistant on day 30
18	S	S	S	R		R (inhA)	
19	S	S	S	R		R (inhA)	
20	S	S	S	R		None	
21	S	S	S	R		R (inhA)	
22	S	S	S	R		R (inhA)	
23	S	S	S	R		R (katG)	
24	S	S	S	R		None	
25	S	S	S	R		R (katG)	

*rpoB *Sequencing was done for one isolate which failed to amplify for MAS-PCR

25/300 isolates (8.3%) had discrepant DST results between MODS and LJ for INH or RIF. R: resistant, S: sensitive, blank (−): no PCR product, blank cells: concordance results between MODS and LJ therefore MAS-PCR was not attempted

Among four isolates with RIF results discrepant between DST-MODS and DST-LJ (Table 2), repeated DST-MODS for RIF 1 μg/ml, MAS-PCR and *rpoB *sequencing confirmed RIF resistance for one isolate (isolates 4, Table 2) and RIF susceptible for three isolates (isolates 1,2,3) (Table 2).

There were two MDR isolates identified by LJ but not by MODS (both isolates were INH resistant and RIF susceptible by MODS, Table 2, isolates 2,4) were resolved by MAS-PCR. Of these two isolates, one isolate was confirmed as RIF resistant by MAS-PCR. The remaining isolate failed to amplify *and rpoB *identified only a synonymous mutation in this gene.

There was one MDR isolate identified by DST-MODS which was INH and RIF susceptible by DST-LJ (Table 2, isolate 1). MAS-PCR detected an INH resistance mutation (*katG315*) in this isolate but no mutation was identified in *rpoB*.

Of 62 isolates with INH resistance by DST-LJ, 17 isolates were INH-susceptible by MODS (Table 3 and Table 2). Of these 17 isolates, resistance mutations in *katG *and *inhA *promoter genes were detected in 35.3% (n = 6/17) and 47.1% (n = 8/17) of isolates, respectively. Three isolates (17.6%, n = 3/17) were wild type by MAS-PCR. Therefore, we concluded that 14/17 isolates were truly INH resistant due to the confirmed presence of resistance mutations. For the remaining 3 isolates we were unable to confirm/exclude resistance since INH resistance conferring mutations may be present outside the MAS-PCR target sites.

**Table 3:** Analysis of MAS-PCR in 62 isolates with INH resistance by proportional DST method, in relation to DST/MODS results

	MAS-PCR						
**DST/MODS**		**KatG**	**inhA**	**KatG and inhA**	**WT**	**Indefinite**	**Total**
	
	**Resistant**	33	03	01	07	01	45
	
	**Sensitive**	06	08	00	03	00	17
	
	**All strains**	39	11	01	10	01	62

Of 17 isolates with INH sensitive by DST/MODS, 8 isolates (47.1%) had a mutation on *inhA *promoter gene and 6 isolates (35.3%) carried amutation on *katG *gene

Twenty-two isolates with INH results discrepant between DST-MODS and DST-LJ were repeated by DST-MODS for INH at a lower concentration (0.1 μg/ml instead of 0.4 μg/ml) because we hypothesised the 0.4 μg/ml concentration may be missing low-level INH resistant isolates since a recent meta-analysis [8] suggests increased sensitivity of the 0.1 μg/ml INH concentration for MODS. Eighteen (81.8%, n = 18/22) samples were culture negative on MODS (smear results were negative for 13 samples, scanty for one sample and 1+ for 4 samples). Therefore, DST results for these samples were not available. The repeat results of the remaining 4 isolates were completely accordant with those of DST-LJ and MAS-PCR; two isolates converted from resistant to susceptible and the remaining two converted from susceptible to resistant. The latter two isolates carried a mutation in the *inhA *promoter region detected by MAS-PCR.

### Analysis of samples with one positive control by DST-MODS (DST-MODS results were recorded as uninterpretable)

Thirty-two DST-MODS samples with one positive control (Figure 1) were analyzed separately. Of which, 21.9% (n = 7/32) were negative by both MGIT and LJ culture and 78.1% (n = 25/32) were positive by either MGIT or LJ culture. Data from routine testing showed that 96% of these samples (n = 24/25) were negative by direct smear and only one sample had a positive smear result (scanty result).

### Time to detection (Median time in days)

#### Time to detection (positive) for TB diagnosis

**Figure 3:**
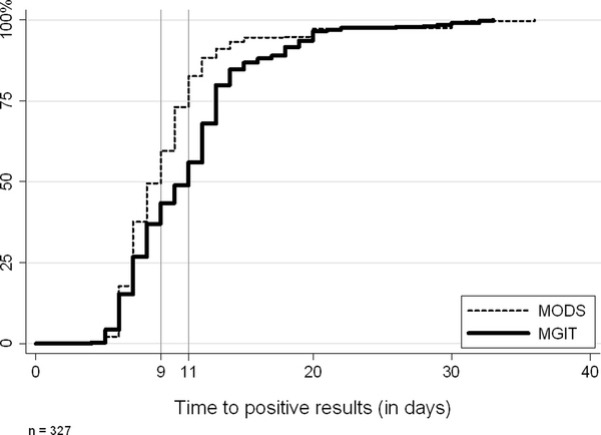
**Time to positive of MODS culture and MGIT culture**. In 327 samples positive by both MODS and MGIT, the turnaround time of MODS and MGIT were 9 days (IQR: 7-11 days) and 11 days (IQR: 7-13 days), *P *< 0.001

Time to positive was recorded as the duration of time from sample inoculation to positive result available in days. For 327 samples positive by both MODS and MGIT, the turn-around time of MODS was 9 days (IQR: 7-11 days) while it was 11 days (IQR: 7-13 days) for MGIT. Compared to MGIT, the MODS results was available faster in 182 (56%), at the same time in 45 (14%) and slower in 100 (31%) samples. The time to positive of MODS was significantly faster than that of MGIT (*P *< 0.001) (Figure 3).

#### Time to DST result available

Time to DST result available was defined as the period of time from sample inoculation to DST result available, in days. For DST-MODS, this duration was exactly the same as the time to positive by MODS culture (median = 9 days). For DST-LJ, this time was the sum of time to positive of MGIT or LJ culture (a median of 11 days) and performing DST-LJ (42 days). Therefore, the median total turn-around time of DST-LJ was 53 days which was much slower than DST-MODS.

### Contamination

Contamination with fungi and cross-contamination were analyzed for all 709 samples.

#### Contamination of MODS culture

The initial fungal contamination rate was 0.6% (n = 4/709). No re-decontamination and re-culture on MODS were attempted for contaminated MODS because of the limited volume of MODS culture (1 ml). Three samples were cross-contaminated with H_37_R_v_, the positive control isolate used in this study and seven samples were suspected of cross-contamination between samples, generating a maximum possible cross-contamination rate of 1.4% (n = 10/709).

#### Contamination of MGIT and LJ

The final fungal contamination rates of MGIT and LJ were 0.7% (n = 5/709) and 0.6% (n = 4/709), respectively. Four samples were contaminated for both MGIT and LJ. Cross-contamination by MGIT and LJ was not determined.

## Discussion and conclusion

Our data shows that MODS is a sensitive and rapid method for diagnosis of TB and MDR-TB. Although the TB detection rate of MODS (50.5%) was not significantly different from MGIT (51.6%, *p *= 0.8) and LJ (44.4%, *p *= 0.2), MODS was faster than MGIT with a median time to detection of 9 days vs 11 days in samples positive by both methods.

For MDR detection, the turnaround time strongly favored DST-MODS (9 days) over DST-LJ (53 days). The agreement, sensitivity, specificity, PPV and NPV of DST-MODS against DST-LJ for detection of MDR-TB isolates were 99%, 77.8%, 99.7%, 87.5% and 99.3%, respectively. The low PPV for MDR detection may be due to the low MDR-TB prevalence in the study population (3.8% as reported in this study by the gold standard DST-LJ method).

The sensitivities in detection of INH and RIF resistance in our study were lower than those from the study of Moore *at el *(72.6% vs 84.6% for INH and 72.7% vs 100% for RIF) [15] although both studies used the same INH concentration (0.4 μg/ml) and RIF concentration (1 μg/ml). These concentrations have been recommended in the MODS guidelines from the MODS development team in Peru [30]. However, a recent meta-analysis published after completion of this study concluded that the sensitivity of INH-resistance detection was higher with a concentration of 0.1 μg/ml without loss of specificity [8]. To address this issue, we attempted to repeat DST-MODS and performed MAS-PCR for 26 isolates with discrepant results between DST-MODS and DST-LJ for INH and RIF.

For INH discrepant isolates, we found that 8/17 (47%) isolates susceptible by DST-MODS but resistant by DST-LJ carried mutation on *inhA *promoter region (Table 2 and Table 3). Previous studies have shown that *inhA *promoter mutation is associated with low-level phenotypic INH resistance (0.2 μg/ml) [31]. We attempted to repeat DST-MODS for INH at 0.1 μg/ml concentration for all of these 8 processed samples but only 2 samples were re-identified as INH resistant. The remaining samples were negative by MODS culture due to low bacterial load. This is a limitation of this technique. Recently, Mello *et al. *found that the sensitivity of DST-MODS for detection of INH resistant isolates increased to 96.7% if INH 0.1 μg/ml was used for the MODS assay [32]; and a similar conclusion was reported from a meta-analysis [8]. This review supported the use of INH 0.1 μg/ml for DST-MODS and our data also supports the conclusion that the use of 0.4 μg/ml reduces sensitivity in comparison with conventional DST. The clinical applicability of these concentrations has not been determined. It is possible that a low-level resistance to INH as defined by current in vitro breakpoints may not translate to clinical resistance. Further research is required to clarify the appropriate management of these patients.

After resolving discrepant results between DST-MODS and DST-LJ for RIF for 4 isolates (Table 2) by repeated DST-MODS, MAS-PCR and *rpoB *sequencing, the final sensitivity of DST-MODS for detection of RIF was 77.8% (n = 7/9). However, this sensitivity is still lower than previous studies [15,32] although the number of RIF resistant isolates in this study was small leading to wide 95% confidence intervals on the sensitivity estimate (39-93%).

For 25 isolates with discrepant DST results between DST-MODS and DST-LJ for INH or RIF (Table 2), 20 isolates were susceptible by DST-MODS for either INH 0.4 μg/ml or RIF 1 μg/ml but resistant by DST-LJ. One probable explanation is that the bacterial load present in processed samples was not equally aliquoted into each of 4 wells of DST-MODS due to the clumping characteristic of M. *tuberculosis*. It is possible higher bacterial concentrations were present in control wells than in the drug-containing wells because processed samples were aliquoted into control wells first and then the drug-containing wells; and therefore cording formation was detected earlier in control wells than in the drug-containing wells if the isolate was resistant. As a result, at the reading time, growth was seen in control wells but not in drug-containing wells and this isolate was determined as a susceptible isolate by DST-MODS. The clumping of bacilli may be the main factor leading to only a single positive control well for 32 samples in our study. Samples with low bacterial load (smear negative, smear scanty and smear 1+) are more likely to result in inconsistent results by direct DST-MODS due to unequal aliquoting.

The only equipment needed to perform the MODS assay are an inverted microscope, tissue culture plate and consumables, biological safety cabinet and incubator. The technical competence required is aseptic technique and microscopy skills, National TB Programmes applying smear already have a workforce of experienced microscopists. A commercial MODS plate (TB MODS kit™) has been developed by Hardy Diagnostics, USA in collaboration with PATH and is under evaluation. MODS is appropriate for screening for MDRTB in high burden countries where such tests are urgently needed.

MODS meets many criteria for an MDR TB diagnostic test applicable for high-burden settings; it is rapid, low-cost and accurate and can be performed without the need for biological safety level 3 laboratories (if the plate is not opened after inoculation). Therefore, MODS is an alternative method for rapid MDR-TB screening in these settings. Recently, wide application of MODS in resource-constrained settings has been endorsed by WHO [9]. However, an international standard operating procedure and a quality assurance system accredited by WHO should be developed to standardize and maintain accuracy.

## Competing interests

The authors declare that they have no competing interests.

## Authors' contributions

DTMH: Conceived, designed, performed, coordinated and analysed the study and prepared the manuscript. NTNL: Conceived, designed the study and contributed to the manuscript. MW: Performed statistical analysis and contributed to the manuscript. VSK: performed molecular analysis and contributed to the data analysis. HTTH: Performed statistical analysis and contributed to the manuscript. NHD: Conceived, designed the study, acquisition and interpretation of the data. TMH: Conceived, designed the study, acquisition and interpretation of the data. VMB: Conceived, designed the study, acquisition and interpretation of the data. NTPT: Conceived, designed the study, acquisition and interpretation of the data. TVQ: Conducted the MODS experiments and interpretation of the data. NTBT: Conducted the MODS experiments and interpretation of the data. VTH: Conducted the MODS experiments and interpretation of the data. NTN: Conceived, designed the study, acquisition and interpretation of the data. DVH: Conceived, designed the study, acquisition and interpretation of the data. PTHA: performed molecular analysis and interpretation of the data. NHD: Conceived, designed the study and contributed to the manuscript. JF: Conceived, designed the study, contributed to the manuscript. MC: Conceived, designed, performed, analysed the study and prepared the manuscript. All authors read and approved the final manuscript.

## Pre-publication history

The pre-publication history for this paper can be accessed here:

http://www.biomedcentral.com/1471-2334/12/49/prepub
